# Isolation, characterization, and genome assembly of *Barnettozyma botsteinii* sp. nov. and novel strains of *Kurtzmaniella quercitrusa* isolated from the intestinal tract of the termite *Macrotermes bellicosus*

**DOI:** 10.1093/g3journal/jkab342

**Published:** 2021-09-29

**Authors:** Gerard Arrey, Guangshuo Li, Robert Murphy, Leandro Guimaraes, Sefa Alizadeh, Michael Poulsen, Birgitte Regenberg

**Affiliations:** Section for Ecology and Evolution, Department of Biology, University of Copenhagen, Copenhagen 1165, Denmark; Section for Ecology and Evolution, Department of Biology, University of Copenhagen, Copenhagen 1165, Denmark; Section for Ecology and Evolution, Department of Biology, University of Copenhagen, Copenhagen 1165, Denmark; Section for Ecology and Evolution, Department of Biology, University of Copenhagen, Copenhagen 1165, Denmark; Section for Ecology and Evolution, Department of Biology, University of Copenhagen, Copenhagen 1165, Denmark; Section for Ecology and Evolution, Department of Biology, University of Copenhagen, Copenhagen 1165, Denmark; Section for Ecology and Evolution, Department of Biology, University of Copenhagen, Copenhagen 1165, Denmark

**Keywords:** *Barnettozyma botsteinii*, d-xylose, insect-associated yeasts, *Kurtzmaniella quercitrusa*, new yeast species

## Abstract

Bioconversion of hemicelluloses into simpler sugars leads to the production of a significant amount of pentose sugars, such as d-xylose. However, efficient utilization of pentoses by conventional yeast production strains remains challenging. Wild yeast strains can provide new industrially relevant characteristics and efficiently utilize pentose sugars. To explore this strategy, we isolated gut-residing yeasts from the termite *Macrotermes bellicosus* collected in Comoé National Park, Côte d’Ivoire. The yeasts were classified through their Internal Transcribed Spacer/Large Subunit sequence, and their genomes were sequenced and annotated. We identified a novel yeast species, which we name *Barnettozyma botsteinii* sp. nov. 1118^T^ (MycoBank: 833563, CBS 16679^T^ and IBT 710) and two new strains of *Kurtzmaniella quercitrusa*: var. *comoensis* (CBS 16678, IBT 709) and var. *filamentosus* (CBS 16680, IBT 711). The two *K. quercitrusa* strains grow 15% faster on synthetic glucose medium than *Saccharomyces cerevisiae* CEN.PK^T^ in acidic conditions (pH = 3.2) and both strains grow on d-xylose as the sole carbon source at a rate of 0.35 h^−1^. At neutral pH, the yeast form *of K. quercitrusa* var. *filamentosus*, but not var. *comoensis*, switched to filamentous growth in a carbon source-dependent manner. Their genomes are 11.0–13.2 Mb in size and contain between 4888 and 5475 predicted genes. Together with closely related species, we did not find any relationship between gene content and ability to grow on xylose. Besides its metabolism, *K. quercitrusa* var. *filamentosus* has a large potential as a production organism, because of its capacity to grow at low pH and to undergo a dimorphic shift.

## Introduction

The biotechnology industry has historically relied on yeast species to carry out fermentation processes (Demain 2000). A major challenge today is the conversion of raw plant biomass directly into organic compounds of added value, such as organic acids or biogas (Qin *et al.* 2017; Martínez-Gutiérrez 2018; Lee *et al.* 2019). Lignocellulose material is mainly composed of cellulose (30–50%), hemicellulose (20–30%), and lignin (15–25%) (Kricka *et al.* 2014). To break down these complex polysaccharides, filamentous fungi, such as *Trichoderma reesi*, *Aspergillus niger* and *Aspergillus oryzae*, and their secreted enzymatic cocktails have been used with success (Mäkelä *et al.* 2014; Lange 2017). Because these fungi do not efficiently ferment pentose sugars such as d-xylose and l-arabinose produced during depolymerization of hemicelluloses, yeast species or genetically engineered *Saccharomyces cerevisiae* strains have been suggested for downstream processes (Bettiga *et al.* 2009). However, co-fermentation of hexoses and pentoses is still a major challenge during the efficient conversion of lignocellulosic plant biomass, mainly due to difficulties in integrating a new catabolic pathway within an existing metabolic network (Young *et al.* 2010). There is therefore a need to identify and optimize yeasts that can efficiently ferment both pentoses and hexoses. One strategy is to isolate novel wild yeast strains that can metabolize these sugars, which can either serve as a production organism on their own or as a genetic resource for metabolic pathways that can be engineered into industrial strains (Steensels *et al.* 2014).

The unicellular yeasts are commonly found in soil, plants, and flowers, and within the microbiome of insects (bees, beetles, termites, and ants), where they establish a variety of relationships, ranging from obligate to facultative mutualisms (Ganter 2006; Greig and Leu 2009; Kurtzman *et al.* 2011c; Buzzini *et al.* 2017). Described yeast species are found both in the Basidiomycota and Ascomycota phyla and are thought to represent only 1% of the expected yeast diversity worldwide (Robert 2006). In addition, yeast species from tropical rainforest, desert, or tundra ecosystems are underrepresented among characterized species (Kurtzman *et al.* 2015; Groenewald *et al.* 2017) and, therefore, serve as a reservoir for novel species with new characteristics. One example is the gut-residing yeasts from the genus of *Candida*, *Pichia*, *Sporothrix*, and *Debratomyces*, which have been isolated from a variety of termite species (Schäfer 1996; Stefanini 2018). Here, we explored yeasts within *Macrotermes bellicosus* termite guts to find novel yeast strains attractive from a biotechnological perspective and to shed light on insect–yeast relationships. *M. bellicosus* is a fungus-growing termite species (sub-family Macrotermitinae) that has an obligate symbiosis with the basidiomycete fungus *Termitomyces* (Basidiomycota: Lyophyllaceae) maintained on plant material harvested by the termites from outside the nest (Rouland-Lefèvre 2000). During a first passage through the termite gut, this plant biomass is mixed with asexual fungal spores and the resulting feces is used to build a so-called fungus comb (Leuthold *et al.* 1989). The maturation of the comb allows the fungus to grow and the plant material to be degraded. Termites will ingest the mature comb and during a second gut passage obtain nutrition (Poulsen *et al.* 2014; da Costa *et al.* 2019). The combined enzymatic activities from the termite, the gut microbiome, and the fungus account for the degradation of up to 90% of all dead wood in Kenya (Buxton 1981). Altogether, their foraging and digestive capabilities substantially contribute to carbon cycling in these ecosystems (Sugimoto *et al.* 2000) converting wood, leaves, and roots (Buxton 1981; Collins 1981; Smith *et al.* 2019).

In this study, we present and characterize two novel strains and one new species of yeast isolated from the gut of *M. bellicosus.* The two novel strains belong to the species *Kurtzmaniella quercitrusa*, phylum Ascomycota, and they were thus named *K. quercitrusa* var. *comoensis* (strain number in this work 1112) and *K. quercitrusa* var. *filamentosus* (strain number in this work 1120). We also identified a previously unknown species from the Ascomycota, which we name *Barnettozyma botsteinii* sp. nov. 1118^T^ (strain number in this work 1118). All three strains assimilate pentose sugars, which make them potentially suitable for biotechnological applications and interesting for further genomic and metabolic studies. Their degradation of plant-derived polymers suggests a potential role in assisting termite and *Termitomyces* metabolism and supports their potential for biotechnological production.

## Methods

### Sample collection

**Figure 1:**
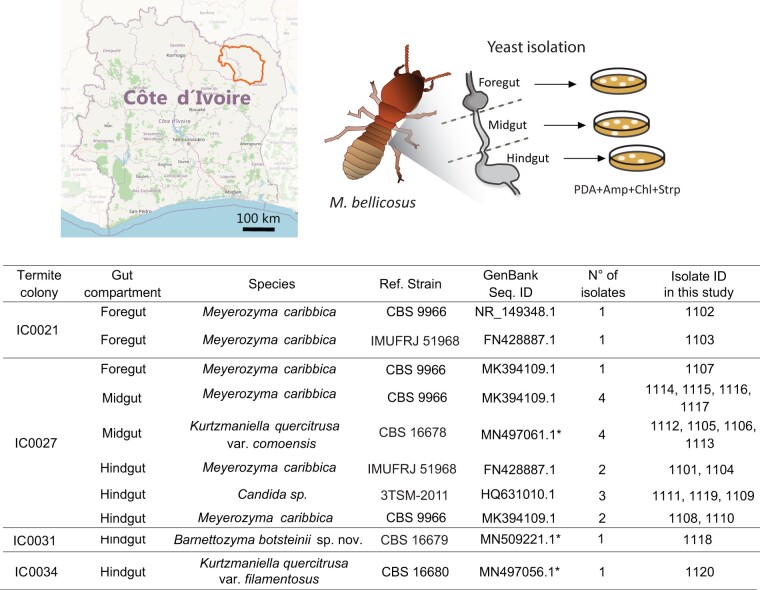
Origin and identification of yeast isolates. Small workers termites were collected in Comoé National Park, in the north east of Côte d’Ivoire (highlighted area in the map) from four *Macrotermes bellicosus* termite colonies (IC0021, IC0027, IC0031, and IC0034) (see Methods). Guts were dissected into foregut, midgut, and hindgut and yeast species were identified by comparing their ITS rDNA sequences to strains in the NCBI GenBank repository. According to their classification and origin, we grouped the isolates and assigned an internal ID. GenBank sequence IDs denoted with * were generated in this study. Otherwise, sequences from the reported isolates were found to be identical to the GenBank IDs provided. Map image modified from © OpenStreetMap contributors.

We collected *M. bellicosus* minor workers from the inside of wild nests in Comoé National Park, Côte d’Ivoire during the month of November 2018. Specifically, the termite nests were located in the proximity of the Comoé National Park Research Station (8°46′11″N, 3°47′21″W), in the southwest end of the park next to the Comoé River. The gut of small workers (25 termites per nest) was dissected *in situ* and divided into midgut, hindgut, and foregut (Figure 1) (Bos *et al.* 2020). Five to ten replicates were pooled together to increase the starting material and manual tissue dissociation was performed with a pestle in sterile 1× phosphate-buffered saline (PBS) supplemented with Tween 80 (5 mg/ml). The resulting lysates were diluted (1/100 and 1/1000) and spread on potato dextrose agar plates. The medium was prepared by diluting 39 g/l of PDA (Sigma Aldrich, 70139) in water, autoclaved, and supplemented with 0.1 g/l of chloramphenicol, 0.05 g/l of streptomycin sulfate and 0.035 g/l of ampicillin before pouring it into plates. Plates were then incubated at 30°C until growth was observed. To screen for yeast species, single colonies were first streak out and subsequently incubated with YPD medium supplemented with 6% ethanol at room temperature until growth was observed. Isolates with a yeast-like morphology under the microscope were then subjected to genomic DNA extraction and rDNA sequencing.

### Internal Transcribed Spacer and D1/D2 sequencing and phylogeny

For genomic DNA (gDNA) extraction, a standardized protocol for yeasts was applied to all the isolates. Briefly, yeast cell wall was broken using glass beads and vortexing. Buffers P1, P2, and N3 (Qiagen, Cat. No.: 19051, 19052, and 19064) were used to isolate the DNA fraction, which was further precipitated by the addition of isopropanol. Finally, after an ethanol wash, pellets were resuspended in TE buffer (10 mM Tris-HCl, pH = 8, 1 mM EDTA) and DNA concentration was assessed by Nanodrop. Primers ITS4 (5′-TCCTCCGCTTATTGATATGC) and ITS1 (5′-CTTGGTCATTTAGAGGAAGTAA) (Kuiper-Goodman and Scott 1989) were used to amplify Internal Transcribed Spacer (ITS) regions while primers NL1 (5′-GCATATCAATAAGCGGAGGAAAAG) and NL4 (5′-GGTCCGTGTTTCAAGACGG) were used to amplify the Large Subunit of the nuclear ribosomal RNA (LSU rRNA) gene region (Kurtzman and Robnett 1997). Both PCR products were purified, Sanger sequenced and results aligned to NCBI database using BLASTn (Altschul *et al.* 1990). Nucleotide sequences for a set of the most closely yeast species were retrieved from GenBank (Supplementary Table S1). Alignment was performed with Mafft v.7.471 (http://mafft.cbrc.jp/alignment/software/) and a maximum-likelihood (ML) phylogeny based on ITS and LSU was constructed in MEGA v. X (Kumar *et al.* 2018). Branch support was obtained from bootstrap analysis with 1000 repetitions.

### Genome sequencing and analyses

We sequenced and assembled the genomes of isolates 1118, 1112, and 1120 in the following steps. Code used is provided in Supplementary File S1.

#### DNA extraction, sequencing, and assembly

Whole-genome sequencing of isolate 1118, 1112, and 1120 was done by first extracting DNA using a scaled-up CTAB extraction (Poulsen *et al.* 2014). Whole-genome sequencing was performed using a combination of 100/150 bp paired-end shotgun (BGIseq/DNBseq) and long-read (PacBio sequel) sequencing by BGI. The resulting sequences were checked for quality using FastQC v0.11.9 (Andrews *et al.* 2012). Genomes were assembled using three different approaches and the best result was chosen for subsequent analyses. First, short and long reads were assembled together and used to generate scaffolds using SPAdes 3.14.1 (Nurk *et al.* 2013). Second, long reads were assembled into scaffolds with Canu 2.0 (Koren *et al.* 2017) and then polished by the short reads using NextPolish 1.3.1 (Hu *et al.* 2020). Finally, short reads were assembled into scaffolds alone, also using SPAdes 3.14.1 (Nurk *et al.* 2013). Contigs that were shorter than 500 bp were discarded in the final assemblies, and assembly quality was assessed by quantifying genome completeness based on the expected gene content of the Benchmarking Universal Single-Copy Orthologs (BUSCO), version 4.4.1, against the database for fungal genomes (Seppey *et al.* 2019).

#### Genome annotation

Genomes were annotated using MAKER v3.01.03 (Holt and Yandell 2011). Two *ab initio* gene predictors were used with the MAKER pipeline: SNAP v2013-11-29 (Leskovec and Sosič 2016) and AUGUSTUS v3.3.3 (Stanke *et al.* 2004), each of which was trained for individual genomes. The training of both SNAP and AUGUSTUS requires pre-existing gene models as training data. Therefore, an initial MAKER analysis was carried out where gene annotations were generated directly from homology evidence without using the *ab initio* gene predictors. The resulting gene annotations supported by homology evidence were then used to train SNAP and AUGUSTUS. Once both *ab initio* gene predictors were trained, they were used together with homology evidence in a full MAKER analysis. Homology evidence was only used to inform gene predictions. Resulting gene models supported by homology evidence were used to re-train SNAP and AUGUSTUS. A second-round analysis was conducted using the newly trained SNAP and AUGUSTUS parameters, and once again the resulting gene models with homology supports were used to re-train SNAP and AUGUSTUS. Finally, a third round of MAKER analysis was performed using the new SNAP and AUGUSTUS parameters. All resulting gene models are reported, and these comprise the final set of annotated genes.

Functional gene annotation was performed using InterProScan version 5.48-83.0 (Jones *et al.* 2014) with annotation of gene ontology (GO) terms (Ashburner *et al.* 2000), KEGG pathways (Kanehisa *et al.* 2021), and Pfam 33 (Mistry *et al.* 2021). The predicted proteins sequences were blasted against NCBI-NR (O’Leary *et al.* 2016) and Swiss-Prot databases (Bateman 2019) with an *E*-value cutoff of 1e-5. Noncoding RNA (ncRNA) genes were identified by BAsic Rapid Ribosomal RNA Predictor v0.9, Barrnap (Seemann 2013) and tRNA genes were predicted using the tRNAScan-SE v2.0.5 (Lowe and Chan 2016) algorithm with default parameters. Repeat sequences were identified and classified using RepeatMasker v4.1.0 (Smit *et al.* 2019). The criterion used for LTR family classification was that the 5′LTR sequences should share at least 80% identity over at least 80% of their length for the same family.

#### Multi-locus-sequence-typing analysis

We performed multi-locus-sequence-typing (MLST) using a set of Universal Single-Copy Orthologous genes for all genomes that were identified with BUSCO v4 (Seppey *et al.* 2019) with default settings and using the fungi_odb10.2019-11-20 dataset. We collated 344 (of 758 potential) genes present in all 48 genomes and generated multi-fasta files of the orthologous genes (Supplementary Table S2). These multi-fasta files were aligned with Clustal Omega v1.2.4 (Sievers *et al.* 2011). Phylogenies were generated using RAxML-NG v0.9.9 (Kozlov *et al.* 2019), employing the –all mode, GTR+G model, and a seed of 2. Branch support based on bootstrapping and transfer distance were obtained, before generating a randomly rooted consensus tree using ASTRAL-Pro v1.12 (Zhang *et al.* 2020).

### Yeast media and growth conditions

Strains were grown and kept on YPD medium (10 g/l of yeast extract, 20 g/l of bacto-peptone, and 20 g/l of glucose) at 30°C. To study growth dynamics and carbon source assimilation, strains were grown and kept on minimal medium (1.6 g/l of yeast nitrogen base without amino acids, 5 g/l of ammonium sulfate with a pH of 6, supplemented with 1% glucose or 1% d-xylose). To check d-xylose assimilation in different concentrations and aeration conditions, single colonies from minimal glucose agar plates were used to inoculate glass-test tubes (max. vol. 35 ml) at an OD_600_ = 0.15, containing minimal medium with 2% or 10% d-xylose at different volumes: 3 ml (high aeration), 7.5 ml (low aeration) or 20 ml (microaeration). The tubes were then kept at 30°C with shaking (150 rpm) for 14 days. To calculate the growth rate in d-xylose and d-glucose medium, starter cultures were prepared by growing single colonies from glucose or xylose minimal medium agar plates into corresponding liquid medium buffered to a pH = 3.2 or pH = 6 overnight at 30°C. The next day, the overnight cultures were used to inoculate 50 ml medium to an OD_600_  ∼ 0.1 in 250 ml baffled Erlenmeyer flasks containing the same buffered medium. For both carbon sources, the medium was buffered to a pH = 3.2 with 0.1 M final concentration of phosphoric buffer (1M KH_2_PO_4_ adjusted to pH = 3.2 with phosphoric acid) or to a pH = 6 with 0.1 M final concentration of potassium phosphate buffer (pH = 6). Cultures were incubated at 30°C and 150 rpm. To calculate each µ, OD_600_ measurements from two different single colonies were used. Each individual OD_600_ measurement was obtained by the average of triplicates. As previously described, colonization patterns were made on semi-solid YPD 0.3% agar plates and cultures were grown for 14 days at room temperature (Kurtzman *et al.* 1991).

### Carbon assimilation

Carbon-source assimilation was assessed using microplates (Kurtzman *et al.* 2011c) using Phenotypic Microarray plates (Biolog, PM1 # 12111 and PM2 # 12112). Briefly, the plates were inoculated with strains 1112, 1118, and 1120 in buffer PM containing Dye mix E to facilitate and improve detection, incubated at 30°C for 144 h and the absorbance measured at 550 nm, as described by the manufacturer. Values at +144 h were used to assess growth on each of the carbon sources. For each time point, background absorbance was subtracted and the fold change (FC) over negative control (no carbon source) was calculated. Signals were assigned to a strong growth (FC >3 at 144 h, +++), intermediate growth (FC >2 and ≤3 at 144 h, ++), weak growth (FC >1 and ≤2), and no growth (FC ≤1). Strains were seeded in duplicate plates in two independent experiments. Mean of the FC was calculated.

### Microscopy

Yeast cells for microscopy were grown overnight in YPD medium at 30°C at 150 rpm. Cells were washed once with distilled sterile water and pellets were re-suspended in water. One to five microliters were mounted in a glass slide with a 0.17 mm thin cover slip. Samples were visualized using a Nikon Eclipse E600 and pictures taken with an Optronics MagnaFire CCD Microscope Camera.

### Xylose metabolism gene analysis

We performed detailed analyses of the genes putatively involved in xylose metabolism by comparing the newly assembled genomes to 27 genomes available from GenBank. We inferred the potential for d-xylose utilization if the following orthologues were present: Xylose reductase (*XYL1*), xylitol dehydrogenase (*XYL2*), and d-xylulokinase (*XYL3*), which translate d-xylose to xylulose-5P, where the xylulose-5P enters the pentose phosphate pathway (PPP). Furthermore, we included genes from the PPP oxidative phase involving the enzymes glucose 6-phosphate dehydrogenase (*ZWF1*), 6-phosphogluconate dehydrogenase (*GND1*), and a nonoxidative phase carried out by d-ribulose-5-phosphate 3-epimerase (*RPE1*), ribose-5-phosphate ketol-isomerase (*RKI1*), transketolase (*TKL1*), and transaldolase (*TAL1*), while phosphoglycerate isomerase (*PGI1*) completes the cycle. We counted the number of copies of the genes encoding these enzymes in the genome and visualized differences across yeasts in a heatmap generated in Excel. We obtained and aligned genes coding for these enzymes from all 27 genomes using Mafft v.7.471 (http://mafft.cbrc.jp/alignment/software/) and subsequently predicted the secondary protein structure using PSIPRED (http://bioinf.cs.ucl.ac.uk/psipred/).

## Results

### Phylogenetic analysis of yeast isolates

We collected worker termites of the species *M. bellicosus* in Comoé National Park (Côte d’Ivoire) and dissected their intestines in foregut, midgut, and hindgut for yeast isolation (Bos *et al.* 2020). We identified a total of 20 individual yeast colonies spanning all three different gut compartments and four independent termite colonies (Figure 1). To identify novel species and subspecies, we next characterized the isolates taxonomically by sequencing the ITS and D1/D2 rDNA regions of LSU and aligning the sequences to GenBank repository using BLASTN (Altschul *et al.* 1990) (Figure 1).

From the set of 20 isolates, 11/20 were assigned to *Meyerozyma caribbica*. Next, 3/20 isolates were assigned to *Candida* sp. TMS-2011 and the rest (6/20 isolates) did not have a clear match with any NCBI sequence, showing similarities between 84.4% and 99% to their respective best matches (Figure 1). From the six uncategorized isolates, four (IDs: 1112, 1113, 1105 and 1106) showed three gaps with *K. quercitrusa* KMC-Y23, isolate 1120 showed one mismatch with *K. quercitrusa* WHFS-3 and isolate 1118 showed 12 gaps and 37 mismatches with *Barnettozyma* sp. EN30S02, leading to a similarity of 91%. Because 1112, 1113, 1105, and 1106 had identical rDNA D1/D2 sequences, we grouped them and selected 1112 as a representative of this group (Figure 1).

**Figure 2:**
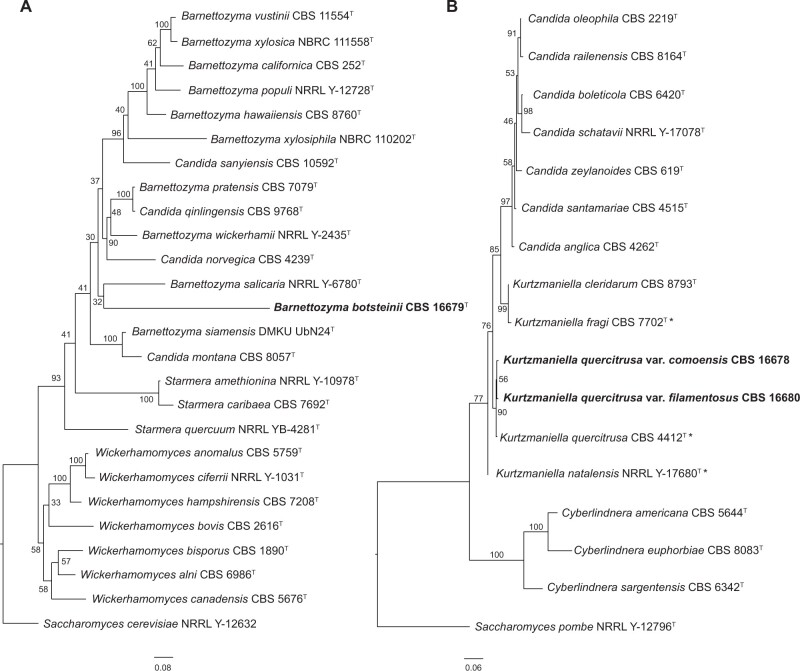
Phylogenetic analyses based on rDNA LSU and ITS sequence alignments. ML phylogenies of the new yeast isolates and their closest species in GenBank (Supplementary Table S1 for sequence IDs) for *B. botsteinii* CBS 16679^T^ (A) and novel strains of *K. quercitrusa* (B). Branches show the result of bootstrap analysis (1000 replicates). The evolutionary distances were calculated using Kimura 2-parameter method and scale bar is set at 0.08 and 0.06 substitutions per site, respectively. The analysis was performed using MEGA v. X (Kumar *et al.* 2018).

The best ML model based on ITS and LSU was TN93+G. The ML tree allowed us to include all closely related yeast species within the genera (Figure 2). *B. botsteinii* sp. nov. (1118) clusters as an independent branch with *Barnettozyma salicaria* NRRL Y-6780^T^ (Figure 2A) while *K. quercitrusa* var. *comoensis* (1112) and var. *filamentosus* (1120) cluster with *K. quercitrusa* type strain CBS 4412^T^ (Figure 2B).

To gain more confidence on its phylogenetic placement, we sequenced the genomes of isolates 1118, 1112, and 1120. We generated a Multiple-Locus-Sequence-Typing (MLST) analysis based on a set of 1706 BUSCO genes inferred to be Ascomycota-specific and single-copy in at least 85% of the available yeast genomes with high (<80%) genome completeness (for general genome features and completeness, see Table 1). The genome sizes and gene content of 1112 and 1120 are similar to *Kurtzmaniella cleridarum*, which has a 12.1 Mb large genome and contains 5478 genes, while 1118 is 11.1 Mb with 4888 genes; comparable to the closest relative with a genome available (*Barnettozyma salicaria*; 11.01 Mb, 5586 genes). The MLST phylogeny thus confirmed the placement of these new isolates in their respective genera and clades (Figure 2).

**Table 1:** Genome characteristics of the three new genomes

Species	Assembly size (Mb)	# contigs	N50 (Mb)	%GC	# genes	# ncRNAs	# Repetitive elements	BUSCO complete	BUSCO duplicated	BUSCO fragmented	BUSCO missing
*B. botsteinii* sp. nov. 1118^T^	11.1	37	1.263	35.0	4888	277	3871	1464	1	55	186
*K. quercitrusa* var. *comoensis*	13.0	12	1.732	39.9	5439	361	2337	1466	13	18	209
*K. quercitrusa* var. *filamentosus*	13.2	17	1.456	45.5	5475	213	3094	1471	4	26	205

BUSCO, Benchmarking Universal Single-Copy Orthologs, see Methods.

### Metabolic profile characterization

To characterize their carbon metabolism, we grew strains 1112, 1120, and 1118 in an array of carbon sources (Supplementary Table S4). Metabolism was detected as a function of respiratory activity (production of NADH) by monitoring the color change in the medium (reduction of tetrazolium dye). From the 190 carbon sources tested, *K. quercitrusa* var. *comoensis* (1112) metabolized among others pentose sugars (2-reoxy-d-ribose, d-arabinose, l-arabinose, d-mannose, d-ribose, l-lyxose, and d-xylose), hexose sugars (α-d-glucose, d-fructose, d-galactose, and d-tagatose), disaccharides (isomaltulose, maltose, sucrose, gentiobiose, palatinose, and turanose), trisaccharides (d-melezitose and maltotriose), organic acids (5-keto-d-gluconic acid, citramalic acid, d,l-malic acid, δ-amino-aleric acid, γ-amino butyric acid, l-malic acid, *N*-acetyl-neuraminic acid succinic acid), Tween (Tween 20, Tween 40, and Tween 80), etc. (see the complete list in Supplementary Table S4).

*K. quercitrusa* var. *filamentosus* (1120) metabolized among others pentose sugars (2-reoxy-d-ribose, d-arabinose, l-arabinose, d-ribose and d-mannose, and l-lyxose), (hexose sugars (α-d-glucose, d-fructose, d-galactose), disaccharides (d-trehalose and gentiobiose), amino acids (l-alanine, l-asparagine, l-aspartic acid, l-glutamine, l-isoleucine, l-lysine, l-proline, and l-pyroglutamic acid), etc. (see the complete list in Supplementary Table S4).

*B. botsteinii* sp. nov. 1118^T^ metabolized among others pentose sugars (2-reoxy-d-ribose, l-arabinose, d-ribose, d-xylose, and l-lyxose), methyl pentose (l-rhamnose), hexose sugars (α-d-glucose, d-glucose-1-Phosphate, d-fructose, d-mannose, and d-psicose), disaccharides (d-cellobiose, gentiobiose, and maltose), trisachharides (maltotriose), sugar alcohols (d-mannitol, d-sorbitol, and glycerol), organic acids (5-keto-d-gluconic acid, α-keto-glutaric acid, acetic acid, citric acid, fumaric acid, pyruvic acid, l-malic acid, and succinic acid), Tween (Tween 20, Tween 40, and Tween 80), etc. (see the complete list in Supplementary Table S4).

We then compared the metabolic profile characterization of the 1118 with other known and closely related *Barnettozyma* and* Candida* species such as *B. salicaria*, *B. siamensis* and *C. montana*. The result revealed that it has different metabolic characteristics, as it can assimilate d-ribose, l-arabinose, gelatine, and maltose, but not salicin and sucrose (Supplementary Table S5). Similarly, the comparison of the physiology of 1112 and *K. quercitrusa* CBS 4412 showed that 1112 differed by being able to assimilate d-arabinose, d-xylose, inulin, l-arabinose, N-acetyl-d-glucosamine and salicin, but not d-glucosamine, d-mannitol, d-trehalose, glycerol, and xylitol. In contrast to *K. quercitrusa* CBS 4412, 1120 grows on d-arabinose, gelatine, l-arabinose and gelatine but not d-mannitol, maltose, sucrose, and l-sorbose (Supplementary Table S5).

### Morphology description

#### Kurtzmaniella quercitrusa *var.* comoensis

**Figure 3:**
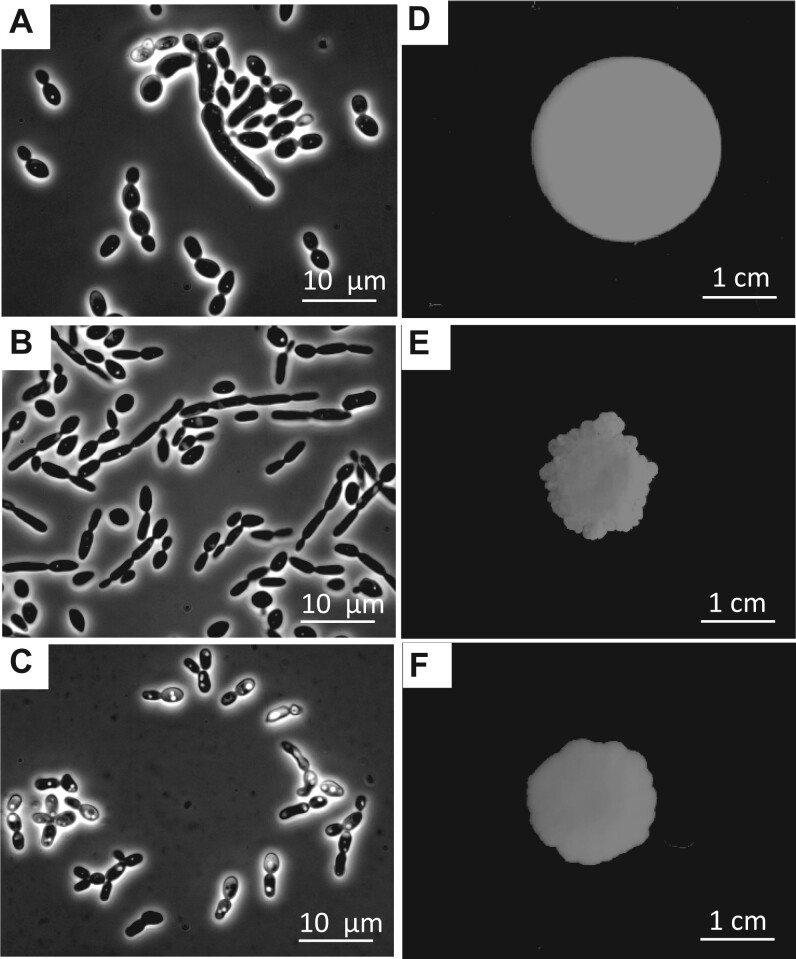
Morphology description. Bright field microscopy pictures (left images) of cells grown in YPD at exponential phase and colonization pattern on YPD 0.3% agar (right images) of *K. quercitrusa* var. *comoensis* (A and D), *K. quercitrusa* var. *filamentosus* (B and E), and *B. botsteinii* sp. nov. 1118^T^ (C and F). Colonies were grown at room temperature for 14 days. Bars show respective scales.

Strain ID in this work: 1112. Microscopic examination of this isolate confirmed that it grew as yeast. Cells appear as black, oval shaped and were 3–4 µm long and 2–3 µm wide during exponential growth in rich (YPD) and minimal glucose medium (see Methods section). Cells divide mitotically by a small bud that fully separates from the mother cell (Figure 3A). Colonies on YPD agar are white, bright, and round, while they appear as a flat and round with an even rim on medium with a low concentration of agar, 0.3% YPD agar (Figure 3D).

#### Kurtzmaniella quercitrusa *var.* filamentosus

**Figure 4:**
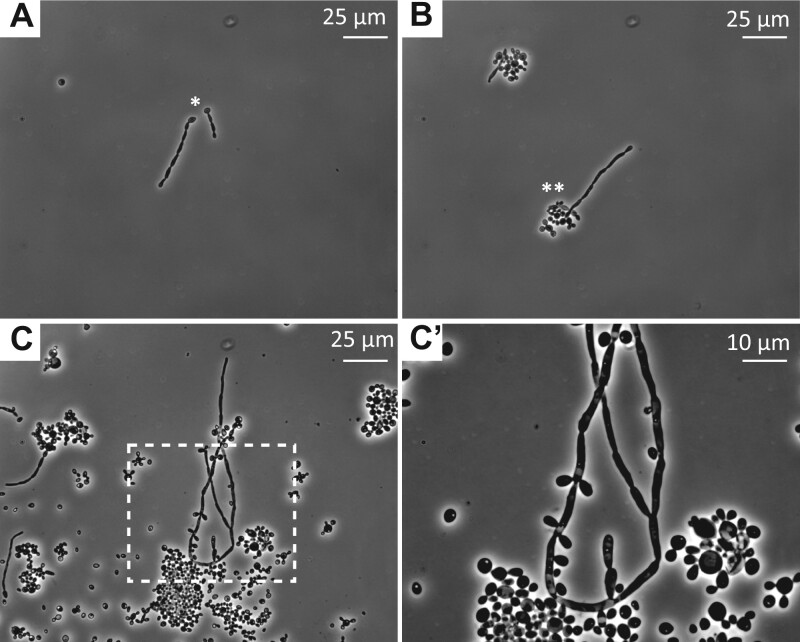
Filamentous growth of *K. quercitrusa* var. *filamentosus* (1120). Cells grown in the synthetic liquid medium 1% xylose (pH = 6) grow as filaments. Single yeast colonies from xylose agar plates were grown in 1% xylose liquid medium buffered at pH = 6 (see Methods). After 48–72 h at 30°C at 150 rpm, small cell chains are visible (A and B) elongating either from single cells (*) or a cluster of cells (**). Eventually, after 96–120 h, these structures develop to longer septate filaments (C and Ć) with bifurcations (arrowhead in Ć). Bright-field microscopy images at indicated scale.

Strain ID in this work: 1120. Yeast cells of this strain are clearly distinguishable from *K. quercitrusa* var. *comoensis*. Being 5–6 µm in length and 1–2 µm wide, cells appear as longer and thinner than 1112 (Figure 3B). Moreover, during exponential growth in glucose-based medium, YPD, and minimal glucose medium (see Methods section), some cells do not fully separate after division leading to formation of 5–6-cell-long pseudohyphal structures. After 5 days of growth in liquid 1% xylose medium, formation of pseudohyphae is strongly enhanced, leading to truly septated hyphae. Hyphae first appear as germ tubes (Figure 4A), which are 2–7 cell long chains growing from a single round cell. They later elongate and mature to true hyphal structures showing bifurcations (Figure 4, C and C**’**). Colonies on rich glucose-based medium (YPD) and minimal glucose medium show a rough, butyrous, and ivory-colored phenotype. In addition, their colonization pattern on 0.3% agar plates is noneven with visible edges and with some growth elevated from the agar (see Methods section) (Figure 3E).

#### Barnettozyma botsteinii *sp. nov.*

*B. botsteinii* sp. nov. 1118^T^ yeast cells are mostly spherical and small (3–4 µm × 2–3 µm) during exponential growth in liquid rich medium (YPD) and minimal glucose media (see Methods section). They are commonly observed as clusters of 3–5 cells forming pseudohyphal structures (Figure 3C). No sign of true hyphae formation was observed in any condition. Although colonies on YPD or minimal glucose medium appear as smooth, the colonization on glucose 0.3% YPD agar leads to an edgy pattern (Figure 3F). The colonies are ivory-colored and flat. The relatively low growth rate observed on liquid medium (Table 2) was also reflected in smaller colonies on solid medium (both on YPD and on SC-1% glucose).

**Table 2:** Growth rates in defined liquid media

	SC-1% glucose				SC-1% xylose			
	pH = 3.2		pH = 6		pH = 3.2		pH = 6	
	**µ_max_ (**± **ST)**	DT	**µ_max_ (**± **ST)**	DT	**µ_max_ (**± **ST)**	DT	**µ_max_ (**± **ST)**	DT
CEN.PK	0.30 (±0.03)	2.3	0.33 (±0.05)	2.1	–	–	–	–
*B. botsteinii* sp. nov*.* 1118^T^	0.03 (±0.005)	25.7	0.24 (±0.02)	2.8	–	–	–	–
*K. quercitrusa* var. nov*.* 1112	0.35 (±0.04)	2.0	0.36 (±0.04)	1.9	–	–	0.036 (±0.001)	19.3
*K. quercitrusa* var. nov. 1120	0.35 (±0.02)	2.0	0.35 (±0.04)	2.0	0.035 (±0.001)	19.6	Hyphae	Hyphae

Growth rate (µ_max_, h^−1^) and doubling times (DT, h) of CEN.PK, *B. botsteinii* sp. nov. 1118, *K. quercitrusa* var. nov. 1112, and *K. quercitrusa* var. nov. 1120 in liquid SC-1% glucose and SC-1% xylose at pH 3.2 or 6. All growth rates are averages of two independent experiments from two independent clones.

### d-Xylose metabolism

**Figure 5:**
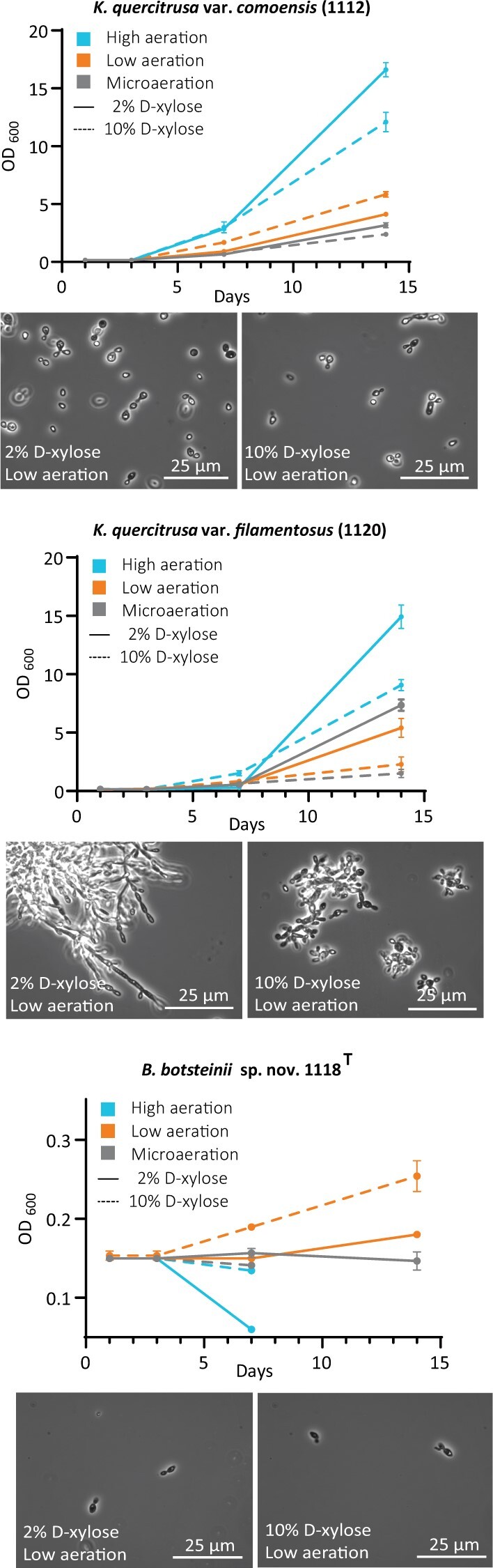
Effect of aeration and sugar concentration on d-xylose growth. OD_600_ measurements at selected times in microaeration (gray), low aeration (orange), and high aeration (blue) in 2% d-xylose (continuous line) or 10% d-xylose (discontinuous line) conditions of *K. quercitrusa* var. *comoensis* (1112) (top), *K. quercitrusa* var. *filamentosus* (1120) (middle), and *B. botsteinii* sp. nov. 1118^T^ (bottom). Representative bright-field microscopy images at indicated scale of cells growing at low aeration (2% or 10% d-xylose) are shown below each graph. Error bars show SD of three OD_600_ measurements. Growth experiments were repeated twice.

After observing that the novel isolates grow on pentose sugars, we next characterized their d-xylose utilization, using different aeration and concentrations of sugar in the growth medium. Strains *K. quercitrusa* var. *comoensis* (strain 1112) and *K. quercitrusa* var. *filamentosus* (strain 1120) show an aeration-dependent growth preference on d-xylose (Figure 5). From the conditions tested, the highest biomass is reached with high aeration in combination with 2% d-xylose, where the OD_600_ after 14 days of growth reaches 16.6 ± 0.6 and 14.9 ± 1.0, respectively. For both *K. quercitrusa* strains, we observed a correlation between aeration levels and OD_600_. A decrease in aeration level correlates with a decrease in OD_600_ both with 2% d-xylose and 10% d-xylose. For example, in low aeration conditions 1112 grows to OD_600_ of 5.8 ± 0.2 with 2% d-xylose and 5.8 ± 0.2 with 10% d-xylose. Similarly, 1120 grows to an OD_600_ of 5.4 ± 0.8 with 2% d-xylose and 2.3 ± 0.6 with 10% d-xylose. We observed a further reduction in the OD_600_ in microaeration conditions, especially in 1112 strain, where it reaches only 3.2 ± 0.29 in 2% d-xylose and 2.4 ± 0.1 in 10% d-xylose.

*B. botsteinii* sp. nov. 1118^T^, on the other hand, did not efficiently grow on d-xylose. Only marginal to very limited growth was observed with microaeration and high d-xylose concentration (Figure 5). Interestingly, *K. quercitrusa* var. *filamentosus* (strain 1120) undergoes a dimorphic shift and forms hyphae in 2% d-xylose medium combined with high or low aeration (Figure 5). The dimorphic shift consisted of formation of pseudohyphae and hyphae of various lengths. No changes in morphology were observed with the others strains.

We next compared the growth rates of *K. quercitrusa* when propagate in d-xylose and d-glucose at low and neutral pH (Table 2). *K. quercitrusa* var. *comoensis* (strain 1112) cells grew in 1% synthetic glucose medium with a doubling time of 2.0 h at pH = 3.2. At pH = 6, the doubling time was similar at 1.9 h. In 1% xylose medium, cells grew at a doubling time of 19.3 h, while they exhibited no growth at pH = 3.2. Cells grew as single yeast cells and divided by budding through exponential phase at both carbon source medium (Table 2). For comparison, a *S. cerevisiae* CEN.PK type strain grew only in synthetic glucose medium at a doubling time of 2.3 h at pH = 3.2 while at pH = 6 it grew with a doubling time of 2.1 h.

*K. quercitrusa* var. *filamentosus* (strain 1120) grew on 1% glucose with a doubling time of 2.0 h at both tested pH (3.2 and 6.0). In xylose, the strain showed pH-dependent morphologic differences (Table 2). At pH = 3.2, the strain grew as single yeast cells with a doubling time of 19.6 h, while at pH = 6.0 medium, pseudohyphae accumulated and matured into true hyphae (Table 2 and Figure 5). Both strains of *K. quercitrusa* (strains 1112 and 1120) showed very similar growth rates in 1% glucose medium and slightly lower than *S. cerevisiae* CEN.PK^T^ strain (Table 2).

*B. botsteinii* sp. nov. 1118^T^ grew in 1% glucose medium at pH = 6 with doubling time of 2.8 h (Table 2). Interestingly, the strain was sensitive to acidic pH that drastically increased its doubling time of 25.7 h at pH = 3.2. Compared to the *K. quercitrusa* strains and *S. cerevisiae* CEN.PK^T^, this species showed the slowest growth rate at both pH (Table 2). Under this experimental growth condition, the strain was not able to grow on 1% xylose medium at either of the tested pH (Table 2).

**Figure 6:**
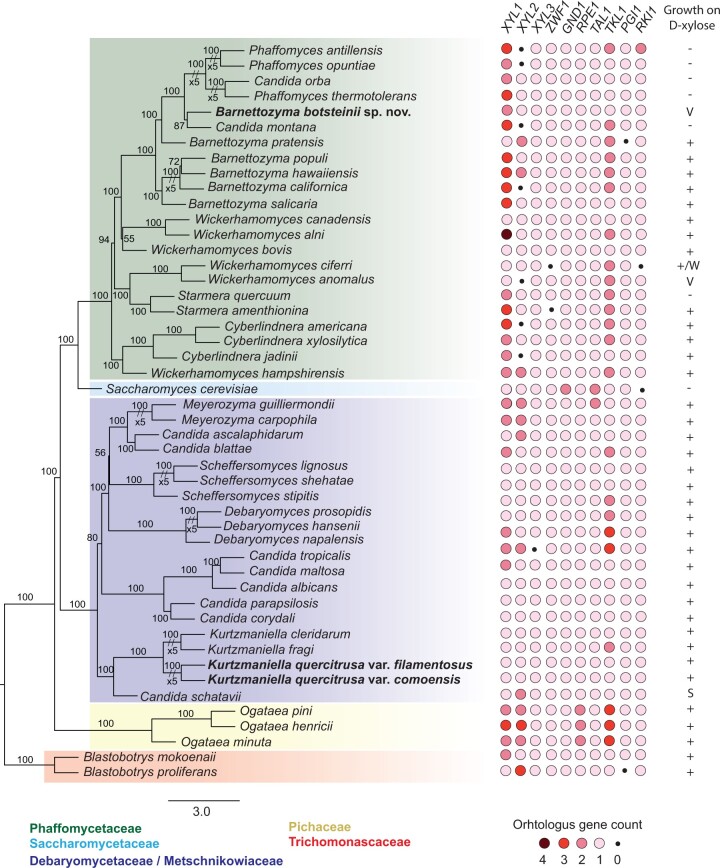
Multiple-locus-sequence-typing analysis. In bold, species and strains described in this study. Consensus ML distance tree based on 344 single-copy orthologous genes with nonparametric bootstrapping with individual gene trees built in RAxML-NG using a GTR+G model. Heatmap of identified number of genes involved in the assimilation and metabolism of xylose across selected yeast species. The heat map indicates that the novel strains and species are comparable to other yeast species (see text for details). Color code indicates number of orthologous genes (top gene panel) from four to none. Positive (+), negative (−), weak (W), variable (V), and slow (S). Details and references of strains used are found in Supplementary Table S6. *The species was transferred from *Candida* to *Kurtzmaniella* genus (Lopes *et al.* 2019).

Copy number changes of genes coding for enzymes in metabolic pathways are known to affect metabolic pathway efflux (Steenwyk and Rokas 2018). Therefore, we investigated the copy number of genes involved in xylose and pentose metabolism. Our comparative genomics analysis for *XYL* and PPP genes revealed that the vast majority of yeasts contained all the orthologues necessary for xylose metabolism (Figure 6). The lack of growth of some of the species on xylose thus does not appear to be as a result of the absence of xylose-metabolizing genes (Figure 6).

### Taxonomy

Description of *B. botsteinii* sp. nov. 1118

MycoBank: MB 833563

*Barnettozyma botsteinii* (bɔt’ʃtaɪni, N.L. gen. n. botsteinii, named after scientist David Botstein) isolated from the hindgut of termite *M. bellicosus* in Côte d’Ivoire. Holotype is preserved at the Westerdijk Institute as CBS 16679^T^ and an isotype at the IBT Culture Collection of Fungi as IBT 710. Cells appear as spherical and small (3–4 µm × 2–3 µm), typically forming clusters of 3–5 cells in pseudohyphal structures. No sign of true hyphae formation was observed in the conditions tested. The colonies appear ivory colored, flat and circular in YPD or minimal 1% glucose medium. However, at low agar (0.3% agar) glucose medium the edge becomes undulate (Figure 3). Cells are able to grow in liquid or solid YPD at 22°C–30°C. The cells grow in 1% glucose medium at pH = 6 with a growth rate of 0.24 h^−^ (±0.02) and doubling time of 2.8 h. Their growth rate is decreased to 0.03 h^−^ (±0.005) corresponding to a doubling time of 25.7 h at pH = 3.2 (Table 2). Based on its genome, phylogeny, and physiology analysis, we confirm that isolate 1118 is clearly distinct from any other known *Barnettozyma* species, we assign the status of sp. nov. to isolate 1118 and thus, propose *B. botsteinii* sp. nov. 1118^T^ as the founder and type strain of a novel yeast species. We name the species after Professor David Botstein, a true inspirer, great geneticist, and important figure for the yeast research community (Botstein *et al.* 1980; Botstein and Fink 1988).

## Discussion

In this study, we isolated novel and biotechnologically relevant yeast strains from termite guts. We describe two novel strains of *K. quercitrusa* and a new species of yeast, which we name *B. botsteinii* based on genomic information*.* The criteria to classify these three novel taxa follows Kurtzman *et al.* (2011c), in which conspecific strains differ by no more than three nucleotides, whereas distinct species show six or more nucleotide differences (Kurtzman *et al.* 2011b).

Here, the novel yeast species *B. botsteinii* sp. nov. 1118^T^ is described based on a single isolate. Novel single-strain fungal taxa descriptions are debated as complete intraspecific variability (including genetic and phenotypic) and its ecological role cannot be fully captured using one strain. However, nearly one-third of described yeast species is based on single isolates (Kurtzman *et al.* 2011a). The documentation and publishing of single strains novel taxa serve as a nucleation point for further studies and the possibility of combining information from strains independently isolated in time. While it was not possible for us to collect new material or find any other available strain with a complete match in GenBank, it is possible that more isolates of *B. botsteinii* sp. nov. are found in the future. This debate was recently reviewed in Brysch-Herzberg *et al.* (2020).

We find that both novel strains of *K. quercitrusa* are able to grow on d-xylose, and their overall metabolic profile makes them interesting for further applied studies. The complex phenotypic change that *K. quercitrusa* var. *filamentosus* (strain 1120) undergoes (filamentation when growing on certain carbon sources) reveals the existence of a metabolic shift that can control alterations in protein secretion and enzyme production similar to that of other yeasts with a dimorphic shift (Gauthier 2017; Celińska and Nicaud 2019).

We identified two novel strains and a new species of yeast by studying a relatively small number of individuals (around 100 termites from only one termite species, *M. bellicosus*). Other yeast strains, without a clear match to described species, have previously been isolated from *Macrotermes subhyalinus* (Yoro *et al.* 2014) and other fungus-growing termites (*Odontotermes formosanus*) (Mathew *et al.* 2012). Therefore, the Macrotermitinae termite sub-family, comprising 330 described species, appears as a potentially extensive biological reservoir for new yeast species and strains of biotechnological relevance.

*K*. *quercitrusa* was first isolated from the insect frass on an oak tree (*Quercus* sp.) (Meyer and Yarrow 1998), type strain CBS 4412^T^. Other isolates of the same species have been collected from other insects (beetles) and from flowers around the world (*e.g.*, Oceania, South America, and South-east Asia) (Crous *et al.* 2004; Molnár *et al.* 2008). Although generally considered nonpathogenic, adult and pediatric human infections have been reported in immunocompromised patients, widening the spectrum of nonalbicans candidiasis (Xiao *et al.* 2014; Westblade *et al.* 2015).

Yeasts are widely used in the biotechnological production of metabolites and protein. However, most do not metabolize pentose sugars well. The two new *K. quercitrusa* strains (var. *comoensis* 1112 and var. *filamentosus* 1120) grow well on hexose sugars, such as glucose, fructose, mannose, and galactose, and are also able to assimilate pentose sugars such as ribose, xylose, arabinose, and lyxose. They show differences in growth on short oligosaccharides in which var. *comoensis* grows on maltose and sucrose while var. *filamentosus* grows on trehalose and adonitol. Furthermore, they both can grow in the presence of complex plant-derived polysaccharides such as mannan, dextrin, and laminarin. The metabolic profile resembles that of *K. quercitrusa*-type strain CBS 4412^T^, including xylose assimilation (Kurtzman *et al.* 2011c). Strain var. *filamentosus* is different to var. *comoensis* in its ability to differentially metabolize both monosaccharides (adonitol) and disaccharides (d-trehalose). The filamentous growth of var. *filamentosus* has been reported in another member of *K. quercitrusa*, strain NRRL Y-5392, although described as pseudohyphae (Kurtzman *et al.* 2011c). In addition, other members of the *Kurtzmaniella* clade, such as *Candida railenensis*, also show hyphae formation under certain conditions (Ramírez and González 1984; Kurtzman *et al.* 2011c). We observed that this phenotype is highly variable and dependent on factors that are common, for example, among *Candida* species, namely: carbon source and concentration, pH, and aeration level (Caplice and Moran 2015).

We also describe a new yeast species, named *B. botsteinii* sp. nov*.* 1118^T^, closely related to *Barnettozyma salicaria* NRRL Y-6780^T^. Metabolically, *B. botsteinii* grows on an array of hexose and pentose sugars. Notably, the strain showed positive growth on short organic acids: acetic and glyoxylic acids (2 carbons), pyruvic acid (3C), fumaric and malic acids (4C), α-ketoglutaric acid (5C), and citric acid (6C). Because many of these are part of the Krebs cycle, this metabolic profile suggests that this species could be classified as Krebs positive (Barnett and Kornberg 1960; Casal *et al.* 2008). The isolated strain grew in culture conditions although at relatively low rates, suggesting that we did not find the best growth conditions for this species. *B. botsteinii* could be dependent on the environment provided by the termite for optimal growth like some other known gut-associated yeasts, such as *Coccidiascus legeri*, which have an obligate relationship with their host insect and are unable to grow on any tested laboratory conditions (Kurtzman *et al.* 2011c; Stefanini 2018).

The annotation and analyses of genes involved in xylose metabolism did not spur differences in the novel strains and other known yeasts, suggesting that the phenotypic variation in growth in this group of yeasts is unlikely to be driven by differences in the gene content. Although no effect of gene copy number was observed, other genetic factors could influence gene expression and explain the observed phenotypic diversity such as single-nucleotide polymorphisms (SNPs), small inversions–deletions, or translocations. In addition, differences in the balance of key metabolic components and co-factors can influence enzyme activities, for example, NADH/NAD+, which are important for xylose metabolism (Riley *et al.* 2016). This suggests that the capacity to assimilate and metabolize xylose may be ancient and conserved in yeasts.

A question that remains open is whether these yeast species stably colonize the termite gut or are ingested during foraging. *K. quercitrusa* strain CBS 4412^T^ was isolated from insect frass and we isolated *K. quercitrusa* var. *filamentosus* from the termite hindgut (*i.e.*, last compartment before faces deposition), suggesting that at least this particular yeast species can survive gut passage, a feature not common to all yeasts nor insect guts (Madden *et al.* 2018). Gut passage is exploited by other yeast species for their dispersal and breeding (Reuter *et al.* 2007; Stefanini *et al.* 2012; Madden *et al.* 2018) and it is possible that *K. quercitrusa* and *B. botsteinii* are also dependent on gut passage for growth, mating, and dispersal.

Biotechnologically, the strains and species characterized here show interesting features that warrant further exploration. *K. quercitrusa* growth on xylose makes them interesting for industrial applications, especially if they are able to co-ferment xylose in the presence of hexoses (Hahn-Hägerdal *et al.* 2007; Ceccato-Antonini *et al.* 2017). Especially, the characteristics of *K. quercitrusa* var. *filamentosus* make it suited for biotechnologically production, since growth at low pH, metabolism of pentose sugars, and a controllable filamentous state are particularly desired traits. In addition, all three strains appear to be able to metabolize polysorbate, also known as Tween. This is a complex molecule formed by polyexyethylenated sorbitol molecules with lauric acid and used as a detergent and surfactant. Other yeasts such as *Candida albicans* or *Candida parapsilosis* sustain growth on Tween as a sole carbon source, a trait considered as a virulence factor as its lipid content triggers the expression of extracellular phospholipases, including *LIP1* (Fu *et al.* 1997; Slifkin 2000).

In addition, other metabolic processes other than pentose consumption are also biotechnologically attractive. The cellobiose respiration by *B. botsteinii* sp. nov. 1118^T^ suggests the presence of ß-glucosidases enzymes (EC 3.2.1.21), which are able to break down the cellobiose molecule into single glucose molecules. During the depolymerization of cellulose, cellobiose is produced which inhibits cellulose-degrading enzymes (cellulose 1,4-ß-cellobiosidase and endoglucanases), reducing the overall efficiency (Murphy *et al.* 2013; Sørensen *et al.* 2013). Thus, the addition of ß-glucosidases to the process can greatly improve its efficiency (Woodward and Wiseman 1982).

In conclusion, the intestine of fungus-growing termites appears to be a promising niche for the identification of new yeast strains and species. The metabolic profiles of the yeasts isolated from termite gut reflect the adaptations to a plant-based diet found either within the wild or within the gut of the termite, and these gut-residing yeasts of *Macrotermes* termites have interesting traits of relevance to the biotechnology industry.

## Data availability

Sanger sequences of D1/D2 rDNA regions of LSU and ITS for isolate 1112, 1120, and 1118 are deposited in the GenBank under the accessions MN497060.1, MN497056.1, MN509221.1, MN497061.1, MN497057.1, and MN509222.1. Genomes of three strains are deposited in GenBank under the BioProject number PRJNA695125, and the accession numbers are JAFIDI000000000, JAFIIU000000000, and JAFIDH000000000. The strains are available as follows: *B. botsteinii* sp. nov. 1118^T^ (MycoBank: 833563, CBS 16679^T^ and IBT 710), *K. quercitrusa* var. *comoensis* 1112 (CBS 16678, IBT 709) and *K. quercitrusa* var. *filamentosus* 1120 (CBS 16680, IBT 711).

Supplementary material is available at figshare: https://doi.org/10.25387/g3.14899278. .

## References

[jkab342-B1] Altschul SF , Gish W, Miller W, Myers EW, Lipman DJ. 1990. Basic local alignment search tool. J Mol Biol. 215:403–410. doi:10.1016/S0022-2836(05)80360-2. 2231712 10.1016/S0022-2836(05)80360-2

[jkab342-B2] Andrews S , Krueger F, Segonds-Pichon A, Biggins L, Krueger C, et al 2012. FastQC: A Quality Control Tool for High Throughput Sequence Data. http://www.bioinformatics.babraham.ac.uk/projects/fastqc.

[jkab342-B3] Ashburner M , Ball CA, Blake JA, Botstein D, Butler H, et al 2000. Gene ontology: tool for the unification of biology. Nat Genet. 25:25–29. doi:10.1038/75556. 10802651 10.1038/75556 PMC3037419

[jkab342-B4] Barnett JA , Kornberg HL. 1960. The utilization by yeasts of acids of the tricarboxylic acid cycle. J Gen Microbiol. 23:65–82. doi:10.1099/00221287-23-1-65. 13796913 10.1099/00221287-23-1-65

[jkab342-B5] Bateman A. 2019. UniProt: a worldwide hub of protein knowledge. Nucleic Acids Res. 47:D506–D515. doi:10.1093/nar/gky1049. 30395287 10.1093/nar/gky1049 PMC6323992

[jkab342-B6] Bettiga M , Bengtsson O, Hahn-Hägerdal B, Gorwa-Grauslund MF. 2009. Arabinose and xylose fermentation by recombinant *Saccharomyces cerevisiae* expressing a fungal pentose utilization pathway. Microb Cell Fact. 8:40.doi:10.1186/1475-2859-8-40. 19630951 10.1186/1475-2859-8-40 PMC2720912

[jkab342-B7] Bos N , Guimaraes L, Palenzuela R, Renelies-Hamilton J, Maccario L, et al 2020. You don’t have the guts: a diverse set of fungi survive passage through *Macrotermes bellicosus* termite guts. BMC Evol Biol. 20:163. doi:10.1186/s12862-020-01727-z. 33297950 10.1186/s12862-020-01727-z PMC7724875

[jkab342-B8] Botstein D , White RL, Skolnick M, Davis RW. 1980. Construction of a genetic linkage map in man using restriction fragment length polymorphisms. Am J Hum Genet. 32:314–331. doi:10.17348/era.9.0.151-162. 6247908 PMC1686077

[jkab342-B9] Botstein D , Fink GR. 1988. Yeast: an experimental organism for modern biology. Science. 240:1439–1443. doi:10.1126/science.3287619. 3287619 10.1126/science.3287619

[jkab342-B10] Brysch-Herzberg M , Wohlmann E, Fischer R. 2020. *Zygosaccharomyces seidelii* sp. nov. a new yeast species from the Maldives, and a revisit of the single-strain species debate. Antonie van Leeuwenhoek. 113:427–436. doi:10.1007/s10482-019 -01352-x. 31721031 10.1007/s10482-019-01352-x

[jkab342-B11] Buxton RD. 1981. Termites and the turnover of dead wood in an arid tropical environment. Oecologia. 51:379–384. doi:10.1007/BF00540909. 28310023 10.1007/BF00540909

[jkab342-B1001] Blackwell M. 2017. Yeasts in Insects and Other Invertebrates. In: Buzzini P., Lachance MA., Yurkov A. (eds) Yeasts in Natural Ecosystems: Diversity. Cham: Springer. 10.1007/978-3-319-62683-3_13

[jkab342-B13] Caplice N , Moran GP. 2015. *Candida albicans* exhibits enhanced alkaline and temperature induction of Efg1-regulated transcripts relative to *Candida dubliniensis*. Genom Data. 6:130–135. doi:10.1016/j.gdata.2015.08.026. 26697354 10.1016/j.gdata.2015.08.026 PMC4664712

[jkab342-B14] Casal M , Paiva S, Queirós O, Soares-Silva I. 2008. Transport of carboxylic acids in yeasts. FEMS Microbiol Rev. 32:974–994. doi:10.1111/j.1574-6976.2008.00128.x. 18759742 10.1111/j.1574-6976.2008.00128.x

[jkab342-B15] Ceccato-Antonini SR , Codato CB, Martini C, Bastos RG, Tauk-Tornisielo SM. 2017. Yeast for pentose fermentation: isolation, screening, performance, manipulation, and prospects. In: Buckeridge M., De Souza A. (eds) Advances of Basic Science for Second Generation Bioethanol from Sugarcane. Cham: Springer. p. 133–157. doi:10.1007/978-3-319-49826-3_8.

[jkab342-B16] Celińska E , Nicaud JM. 2019. Filamentous fungi-like secretory pathway strayed in a yeast system: peculiarities of *Yarrowia lipolytica* secretory pathway underlying its extraordinary performance. Appl Microbiol Biotechnol. 103:39–52. doi:10.1007/s00253-018-9450-2. 30353423 10.1007/s00253-018-9450-2 PMC6311201

[jkab342-B17] Collins NM. 1981. The role of termites in the decomposition of wood and leaf litter in the Southern Guinea savanna of Nigeria. Oecologia. 51:389–399. doi:10.1007/BF00540911. 28310025 10.1007/BF00540911

[jkab342-B18] Crous PW , Gams W, Stalpers JA, Robert V, Stegehuis G. 2004. MycoBank: an online initiative to launch mycology into the 21st century. Stud Mycol. 50:19–22.

[jkab342-B19] da Costa RR , Hu H, Li H, Poulsen M. 2019. Symbiotic plant biomass decomposition in Fungus-Growing termites. Insects. 10: 87. doi:10.3390/insects10040087. 30925664 10.3390/insects10040087 PMC6523192

[jkab342-B21] Demain AL. 2000. Microbial biotechnology. Trends Biotechnol. 18:26–31. doi:10.1016/S0167-7799(99)01400-6 10631778 10.1016/s0167-7799(99)01400-6

[jkab342-B22] Fu Y , Ibrahim AS, Fonzi W, Zhou X, Ramos CF, et al 1997. Cloning and characterization of a gene (LIP1) which encodes a lipase from the pathogenic yeast *Candida albicans*. Microbiology (Reading). 143(Pt 2):331–340. doi:10.1099/00221287-143-2-331. 9043110 10.1099/00221287-143-2-331

[jkab342-B23] Gauthier GM. 2017. Fungal dimorphism and virulence: molecular mechanisms for temperature adaptation, immune evasion, and *in vivo* survival. Mediators Inflamm. 2017:8491383. doi:10.1155/2017/8491383. 28626345 10.1155/2017/8491383 PMC5463121

[jkab342-B24] Groenewald M , Boundy-Mills K, Čadež N, Endoh R, Jindamorakot S, et al 2017. Census of yeasts isolated from natural ecosystem and conserved in worldwide collections. In: Yeasts in Natural Ecosystems: Diversity. 455–476. doi:10.1007/978-3-319-62683-3_15.

[jkab342-B25] Hahn-Hägerdal B , Karhumaa K, Fonseca C, Spencer-Martins I, Gorwa-Grauslund MF. 2007. Towards industrial pentose-fermenting yeast strains. Appl Microbiol Biotechnol. 74:937–953. doi:10.1007/s00253-006-0827-2. 17294186 10.1007/s00253-006-0827-2

[jkab342-B26] Holt C , Yandell M. 2011. MAKER2: an annotation pipeline and genome-database management tool for second-generation genome projects. BMC Bioinformatics. 12:491.doi:10.1186/1471-2105-12-491. 22192575 10.1186/1471-2105-12-491 PMC3280279

[jkab342-B27] Hu J , Fan J, Sun Z, Liu S. 2020. NextPolish: a fast and efficient genome polishing tool for long-read assembly. Bioinformatics. 36:2253–2255. doi:10.1093/bioinformatics/btz891. 31778144 10.1093/bioinformatics/btz891

[jkab342-B28] Jones P , Binns D, Chang HY, Fraser M, Li W, et al 2014. InterProScan 5: genome-scale protein function classification. Bioinformatics. 30:1236–1240. doi:10.1093/bioinformatics/btu031. 24451626 10.1093/bioinformatics/btu031 PMC3998142

[jkab342-B29] Kanehisa M , Furumichi M, Sato Y, Ishiguro-Watanabe M, Tanabe M. 2021. KEGG: integrating viruses and cellular organisms. Nucleic Acids Res. 49:D545–D551. doi:10.1093/nar/gkaa970. 33125081 10.1093/nar/gkaa970 PMC7779016

[jkab342-B30] Koren S , Walenz BP, Berlin K, Miller JR, Bergman NH, et al 2017. Canu: scalable and accurate long-read assembly via adaptive κ-mer weighting and repeat separation. Genome Res. 27:722–736. doi:10.1101/gr.215087.116. 28298431 10.1101/gr.215087.116 PMC5411767

[jkab342-B31] Kozlov AM , Darriba D, Flouri T, Morel B, Stamatakis A. 2019. RAxML-NG: a fast, scalable and user-friendly tool for maximum likelihood phylogenetic inference. Bioinformatics. 35:4453–4455. doi:10.1093/bioinformatics/btz305. 31070718 10.1093/bioinformatics/btz305 PMC6821337

[jkab342-B32] Kricka W , Fitzpatrick J, Bond U. 2014. Metabolic engineering of yeasts by heterologous enzyme production for degradation of cellulose and hemicellulose from biomass: a perspective. Front Microbiol. 5:174. doi:10.3389/fmicb.2014.00174. 24795706 10.3389/fmicb.2014.00174 PMC4001029

[jkab342-B33] Kuiper-Goodman T , Scott PM. 1989. Risk assessment of the mycotoxin ochratoxin A. In: *Biomedical and Environmental Sciences: BES*, p. 179–248. doi:10.1016/B978-0-12-372180-8.50042-1. 2692617

[jkab342-B34] Kumar S , Stecher G, Li M, Knyaz C, Tamura K. 2018. MEGA X: molecular evolutionary genetics analysis across computing platforms. Mol Biol Evol. 35:1547–1549. doi:10.1093/molbev/msy096. 29722887 10.1093/molbev/msy096 PMC5967553

[jkab342-B35] Kurtzman CP , Fell JW, Boekhout T. 2011a. Definition, classification and nomenclature of the yeasts. In: Kurtzman CP, Fell JW, Boekhout T. (eds). The Yeasts, Vol. 1. Elsevier. p. 3–5. doi:10.1016/B978-0-444-52149-1.00001-X.

[jkab342-B36] Kurtzman CP , Fell JW, Boekhout T. 2011b. Gene sequence analyses and other DNA-based methods for yeast species recognition. In: Kurtzman CP, Fell JW, Boekhout T. (eds). The Yeasts, Vol. 1. Elsevier. p. 137–144. doi:10.1016/B978-0-444-52149-1.00010-0.

[jkab342-B37] Kurtzman CP , Fell JW, Boekhout T, Robert V. 2011c. Methods for isolation, phenotypic characterization and maintenance of yeasts. In: Kurtzman CP, Fell JW, Boekhout T, Robert V. (eds). The Yeasts, Vol. 1. Elsevier. p. 87–110. doi:10.1016/B978-0-444-52149-1.00007-0.

[jkab342-B38] Kurtzman CP , Guthrie C, Fink GR. 1991. Guide to yeast genetics and molecular biology. Methods Enzymol. 194:1–863. doi:10.2307/3760517. 2005781

[jkab342-B39] Kurtzman CP , Mateo RQ, Kolecka A, Theelen B, Robert V, et al 2015. Advances in yeast systematics and phylogeny and their use as predictors of biotechnologically important metabolic pathways. FEMS Yeast Res. Sep 15(6):fov050. doi:10.1093/femsyr/fov050. 10.1093/femsyr/fov050 26136514

[jkab342-B40] Kurtzman CP , Robnett CJ. 1997. Identification of clinically important ascomycetous yeasts based on nucleotide divergence in the 5’ end of the large-subunit (26S) ribosomal DNA gene. J Clin Microbiol. 35:1216–1223. doi:10.1128/jcm.35.5.1216-1223.1997. 9114410 10.1128/jcm.35.5.1216-1223.1997 PMC232732

[jkab342-B41] Lange L. 2017. Fungal enzymes and yeasts for conversion of plant biomass to bioenergy and high-value products. In: Heitman, J., Howlett, B., Crous, P. W., Stukenbrock, E., James, T., & Gow, N. A. R. (Eds.) The Fungal Kingdom. American Society of Microbiology. p. 1029–1048. doi:10.1128/microbiolspec.funk-0007-2016. 10.1128/microbiolspec.funk-0007-2016 PMC11687429 28155810

[jkab342-B42] Lee SY , Sankaran R, Chew KW, Tan CH, Krishnamoorthy R, et al 2019. Waste to bioenergy: a review on the recent conversion technologies. BMC Energy. 1:4. doi:10.1186/s42500-019-0004-7.

[jkab342-B43] Leskovec J , Sosič R. 2016. SNAP: A general-purpose network analysis and graph-mining library. ACM Trans Intell Syst Technol. 8:1–20. doi:10.1145/2898361. 28344853 10.1145/2898361 PMC5361061

[jkab342-B44] Leuthold RH , Badertscher S, Imboden H. 1989. The inoculation of newly formed fungus comb with Termitomyces in Macrotermes colonies (Isoptera, Macrotermitinae). Ins Soc. 36:328–338. doi:10.1007/BF02224884.

[jkab342-B45] Lopes MR , Santos ARO, Moreira JD, Santa-Brígida R, Martins MB, et al 2019. *Kurtzmaniella hittingeri* f.a., sp. nov., isolated from rotting wood and fruits, and transfer of three Candida species to the genus Kurtzmaniella as new combinations. Int J Syst Evol Microbiol. 69:1504–1508. doi:10.1099/ijsem.0.003337. 30856091 10.1099/ijsem.0.003337

[jkab342-B46] Lowe TM , Chan PP. 2016. tRNAscan-SE On-line: integrating search and context for analysis of transfer RNA genes. Nucleic Acids Res. 44:W54–W57. doi:10.1093/nar/gkw413. 27174935 10.1093/nar/gkw413 PMC4987944

[jkab342-B47] Madden AA , Epps MJ, Fukami T, Irwin RE, Sheppard J, et al 2018. The ecology of insect – Yeast relationships and its relevance to human industry. Proc Biol Sci. 285:20172733.doi:10.1098/rspb.2017.2733. 29563264 10.1098/rspb.2017.2733 PMC5897634

[jkab342-B48] Mäkelä MR , Donofrio N, De Vries RP. 2014. Plant biomass degradation by fungi. Fungal Genet Biol. 72:2–9. doi:10.1016/j.fgb.2014.08.010. 25192611 10.1016/j.fgb.2014.08.010

[jkab342-B49] Martínez-Gutiérrez E. 2018. Biogas production from different lignocellulosic biomass sources: advances and perspectives. Biotech. 8:233.doi:10.1007/s13205-018-1257-4. 10.1007/s13205-018-1257-4 PMC5928011 29725572

[jkab342-B50] Mathew GM , Ju YM, Lai CY, Mathew DC, Huang CC. 2012. Microbial community analysis in the termite gut and fungus comb of *Odontotermes formosanus*: the implication of Bacillus as mutualists. FEMS Microbiol Ecol. 79:504–517. doi:10.1111/j.1574-6941.2011.01232.x. 22092951 10.1111/j.1574-6941.2011.01232.x

[jkab342-B51] Meyer SA , Yarrow D. 1998. Validation of the names of three candida species. 66:99–101.

[jkab342-B52] Mistry J , Chuguransky S, Williams L, Qureshi M, Salazar GA, et al 2021. Pfam: the protein families database in 2021. Nucleic Acids Res. 49:D412–D419. doi:10.1093/nar/gkaa913. 33125078 10.1093/nar/gkaa913 PMC7779014

[jkab342-B53] Molnár O , Wuczkowski M, Prillinger H. 2008. Yeast biodiversity in the guts of several pests on maize; comparison of three methods: classical isolation, cloning and DGGE. Mycol Progress. 7:111–123. doi:10.1007/s11557-008-0558-0.

[jkab342-B54] Murphy L , Bohlin C, Baumann MJ, Olsen SN, Sørensen TH, et al 2013. Product inhibition of five *Hypocrea jecorina* cellulases. Enzyme Microb Technol. 52:163–169. doi:10.1016/j.enzmictec.2013.01.002. 23410927 10.1016/j.enzmictec.2013.01.002

[jkab342-B55] Nurk S , Bankevich A, Antipov D, Gurevich A, Korobeynikov A, et al 2013. Assembling genomes and mini-metagenomes from highly chimeric reads. Lect Notes Comput Sci. 7821:158–170. doi:10.1007/978-3-642-37195-0_13. 10.1089/cmb.2013.0084 PMC3791033 24093227

[jkab342-B56] O'Leary NA , Wright MW, Brister JR, Ciufo S, Haddad D, et al 2016. Reference sequence (RefSeq) database at NCBI: current status, taxonomic expansion, and functional annotation. Nucleic Acids Res. 44:D733–D745. doi:10.1093/nar/gkv1189. 26553804 10.1093/nar/gkv1189 PMC4702849

[jkab342-B57] Poulsen M , Hu H, Li C, Chen Z, Xu L, et al 2014. Complementary symbiont contributions to plant decomposition in a fungus-farming termite. Proc Natl Acad Sci USA. 111:14500–14505. doi:10.1073/pnas.1319718111. 25246537 10.1073/pnas.1319718111 PMC4209977

[jkab342-B58] Qin L , Li W-C, Zhu J-Q, Li B-Z, Yuan Y-J. 2017. Hydrolysis of Lignocellulosic Biomass to Sugars. In: Fang Z., Smith, Jr. R., Qi X. (eds). Production of Platform Chemicals from Sustainable Resources. Biofuels and Biorefineries. Singapore: Springer. doi:10.1007/978-981-10-4172-3_1.

[jkab342-B59] Ramírez C , González A. 1984. Two new amycelial Candida isolated from decayed wood in the evergreen rainy Valdivian forest of southern Chile. Mycopathologia. 88:99–103. doi:10.1007/BF00436438.

[jkab342-B60] Reuter M , Bell G, Greig D. 2007. Increased outbreeding in yeast in response to dispersal by an insect vector. Curr Biol. 17:R81–R83. doi:10.1016/j.cub.2006.11.059. 17276903 10.1016/j.cub.2006.11.059

[jkab342-B61] Riley R , Haridas S, Wolfe KH, Lopes MR, Hittinger CT, et al 2016. Comparative genomics of biotechnologically important yeasts. Proc Natl Acad Sci USA. 113:9882–9887. doi:10.1073/pnas.1603941113. 27535936 10.1073/pnas.1603941113 PMC5024638

[jkab342-B62] Robert V , Stalpers J, Boekhout T, Tan S. 2006. Yeast biodiversity and culture collections. In: Péter G., Rosa C. (eds). Biodiversity and Ecophysiology of Yeasts. The Yeast Handbook. Berlin, Heidelberg: Springer. 10.1007/3-540-30985-3_3

[jkab342-B63] Rouland-Lefèvre C. 2000. Symbiosis with fungi. In: Abe T., Bignell D.E., Higashi M. (eds). Termites: Evolution, Sociality, Symbioses, Ecology. Dordrecht: Springer. p. 289–306. doi:10.1007/978-94-017-3223-9_14.

[jkab342-B64] Schäfer A , Konrad R, Kuhnigk T, Kämpfer P, Hertel H, et al 1996. Hemicellulose-degrading bacteria and yeasts from the termite gut. J Appl Bacteriol. 80:471–478. doi:10.1111/j.1365-2672.1996.tb03245.x. 9072518 10.1111/j.1365-2672.1996.tb03245.x

[jkab342-B65] Seppey M , Manni M, Zdobnov EM. 2019. BUSCO: assessing genome assembly and annotation completeness. Methods Mol Biol. 1962:227–245. doi:10.1007/978-1-4939-9173-0_14. 31020564 10.1007/978-1-4939-9173-0_14

[jkab342-B66] Sievers F , Wilm A, Dineen D, Gibson TJ, Karplus K, et al 2011. Fast, scalable generation of high-quality protein multiple sequence alignments using Clustal Omega. Mol Syst Biol. 7:539.doi:10.1038/msb.2011.75. 21988835 10.1038/msb.2011.75 PMC3261699

[jkab342-B67] Slifkin M. 2000. Tween 80 opacity test responses of various candida species. J Clin Microbiol. 38:4626–4628. doi:10.1128/jcm.38.12.4626-4628.2000. 11101607 10.1128/jcm.38.12.4626-4628.2000 PMC87648

[jkab342-B68] Smit AFA , Hubley R, Green P. 2019. *RepeatMasker Open-4.1.0*. http://www.repeatmasker.org/

[jkab342-B69] Smith SW , Speed JDM, Bukombe J, Hassan SN, Lyamuya RD, et al 2019. Litter type and termites regulate root decomposition across contrasting savanna land-uses. Oikos. 128:596–607. doi:10.1111/oik.05697.

[jkab342-B70] Sørensen A , Lübeck M, Lübeck PS, Ahring BK. 2013. Fungal beta-glucosidases: a bottleneck in industrial use of lignocellulosic materials. Biomolecules. 3:612–631. doi:10.3390/biom3030612. 24970184 10.3390/biom3030612 PMC4030957

[jkab342-B71] Stanke M , Steinkamp R, Waack S, Morgenstern B. 2004. AUGUSTUS: a web server for gene finding in eukaryotes. Nucleic Acids Res. 32:W309–W312. doi:10.1093/nar/gkh379. 15215400 10.1093/nar/gkh379 PMC441517

[jkab342-B72] Steensels J , Snoek T, Meersman E, Nicolino MP, Voordeckers K, et al 2014. Improving industrial yeast strains: exploiting natural and artificial diversity. FEMS Microbiol Rev. 38:947–995. doi:10.1111/1574-6976.12073. 24724938 10.1111/1574-6976.12073 PMC4293462

[jkab342-B73] Steenwyk JL , Rokas A. 2018. Copy number variation in fungi and its implications for wine yeast genetic diversity and adaptation. Front Microbiol. 9:288.doi:10.3389/fmicb.2018.00288. 29520259 10.3389/fmicb.2018.00288 PMC5826948

[jkab342-B74] Stefanini I. 2018. Yeast-insect associations: it takes guts. Yeast. 35:315–330. doi:10.1002/yea.3309. 29363168 10.1002/yea.3309 PMC5947625

[jkab342-B75] Stefanini I , Dapporto L, Legras JL, Calabretta A, Di Paola M, et al 2012. Role of social wasps in *Saccharomyces cerevisiae* ecology and evolution. Proc Natl Acad Sci USA. 109:13398–13403. doi:10.1073/pnas.1208362109. 22847440 10.1073/pnas.1208362109 PMC3421210

[jkab342-B76] Sugimoto A , Bignell DE, MacDonald JA. 2000. Global impact of Termites on the Carbon Cycle and Atmospheric Trace Gases. In: Abe T., Bignell D.E., Higashi M. (eds) Termites: Evolution, Sociality, Symbioses, Ecology. Dordrecht: Springer. p. 409–435. doi:10.1007/978-94-017-3223-9_19.

[jkab342-B77] Seemann T.. 2013. Barrnap 0.2 - Bacterial Ribosomal RNA Predictor. Barrnap(v 0.9): https://github.com/tseemann/barrnap

[jkab342-B78] Westblade LF , Rostad CA, Hilinski JA, Stanley T, Jerris RC, et al 2015. *Candida quercitrusa* Candidemia in a 6-year-old child. J Clin Microbiol. 53:2785–2787. doi:10.1128/JCM.03657-14. 26063864 10.1128/JCM.03657-14 PMC4508417

[jkab342-B79] Woodward J , Wiseman A. 1982. Fungal and other β-d-glucosidases - Their properties and applications. Enzyme .Microb. Technol., Vol 4. p. 73–79. doi:10.1016/0141-0229(82)90084-9.

[jkab342-B80] Xiao M , Wang H, Lu J, Chen SCA, Kong F, et al 2014. Three clustered cases of candidemia caused by *Candida quercitrusa* and mycological characteristics of this novel species. J Clin Microbiol. 52:3044–3048. doi:10.1128/JCM.00246-14. 24696025 10.1128/JCM.00246-14 PMC4136143

[jkab342-B81] Yoro DT , N'Guessan KF, Dabonne S, Kouassi NK, Koffi-Nevry R, et al 2014. Yeast diversity in the intestinal tract of the fungus-growing termite *Macrotermes subhyalinus*. Int J Adv Res. 2:139.

[jkab342-B82] Young E , Lee SM, Alper H. 2010. Optimizing pentose utilization in yeast: the need for novel tools and approaches. Biotechnol Biofuels. 3:24.doi:10.1186/1754-6834-3-24. 21080929 10.1186/1754-6834-3-24 PMC2993683

[jkab342-B83] Zhang C , Scornavacca C, Molloy EK, Mirarab S. 2020. ASTRAL-Pro: quartet-based species-tree inference despite paralogy. Mol Biol Evol. 37:3292–3307. doi:10.1093/molbev/msaa139. 32886770 10.1093/molbev/msaa139 PMC7751180

